# Methicillin-Resistant *Staphylococcus aureus* Colonization of the Groin and Risk for Clinical Infection among HIV-infected Adults

**DOI:** 10.3201/eid1904.121353

**Published:** 2013-04

**Authors:** Philip J. Peters, John T. Brooks, Sigrid K. McAllister, Brandi Limbago, H. Ken Lowery, Gregory Fosheim, Jodie L. Guest, Rachel J. Gorwitz, Monique Bethea, Jeffrey Hageman, Rondeen Mindley, Linda K. McDougal, David Rimland

**Affiliations:** Centers for Disease Control and Prevention, Atlanta, Georgia, USA (P.J. Peters, J.T. Brooks, S.K. McAllister, B. Limbago, G. Fosheim, R.J. Gorwitz, J. Hageman, L.K. McDougal);; Veterans Affairs Medical Center, Atlanta (H.K. Lowery, J.L. Guest, M. Bethea, R. Mindley, D. Rimland);; Emory University School of Medicine, Atlanta (D. Rimland)

**Keywords:** HIV, methicillin-resistant Staphylococcus aureus, colonization, viruses, bacteria, Georgia, MRSA, staphylococci

## Abstract

Data on the interaction between methicillin-resistant *Staphylococcus aureus* (MRSA) colonization and clinical infection are limited. During 2007–2008, we enrolled HIV-infected adults in Atlanta, Georgia, USA, in a prospective cohort study. Nares and groin swab specimens were cultured for *S. aureus* at enrollment and after 6 and 12 months. MRSA colonization was detected in 13%–15% of HIV-infected participants (n = 600, 98% male) at baseline, 6 months, and 12 months. MRSA colonization was detected in the nares only (41%), groin only (21%), and at both sites (38%). Over a median of 2.1 years of follow-up, 29 MRSA clinical infections occurred in 25 participants. In multivariate analysis, MRSA clinical infection was significantly associated with MRSA colonization of the groin (adjusted risk ratio 4.8) and a history of MRSA infection (adjusted risk ratio 3.1). MRSA prevention strategies that can effectively prevent or eliminate groin colonization are likely necessary to reduce clinical infections in this population.

Methicillin-resistant *Staphylococcus aureus* (MRSA) infections are a substantial cause of illness and a major public health problem (*1*). Although MRSA was traditionally considered a health care–associated pathogen, it has emerged worldwide as a notable cause of community-associated skin and soft tissue infections (*2*). In the United States, MRSA pulsed-field gel electrophoresis (PFGE) type USA300 strains have caused most community-associated MRSA infections (*3*). High rates of community-associated (*4*–*6*) and health care–associated MRSA infections have also been described among HIV-infected persons (*7*), although the underlying basis for this association is unknown. Proposed mechanisms include immune dysfunction (*5*,*7*,*8*), behavioral risk factors (*9*), and increased exposure to the health care system (*10*). The prevalence of MRSA colonization among HIV-infected persons is also high (10%–17%) (*11*,*12*), compared with that in the general US population (0.8%–1.5%) (*13*,*14*). Colonization with *S. aureus* is a risk factor for subsequent clinical infection (*15*,*16*), and the site of colonization may also be an key risk factor (*17*). For example, although the anterior nares is considered the primary reservoir of *S. aureus* (*18*), MRSA PFGE type USA300 might preferentially colonize the buttocks, genitals, and perineum (*17*), leading to more infections in these anatomical areas. Improving our understanding of the interaction between MRSA colonization and clinical infection among persons with HIV is necessary so that effective prevention strategies can be developed for this population.

## Methods

### Study Design

Study participants were recruited from the Atlanta Veterans Affairs Medical Center (Atlanta, GA, USA) HIV clinic, which provides medical care to ≈1,400 HIV-infected veterans and is the largest Veterans Affairs Medical Center HIV clinic in the United States. This study was approved by institutional review boards for Emory University and the Centers for Disease Control and Prevention (CDC) and the Veterans Affairs Research and Development Committee. Eligible participants were HIV-infected, >18 years of age, receiving outpatient medical care at the Atlanta Veterans Affairs Medical Center HIV clinic, and competent to provide informed consent. All eligible participants who attended the clinic from September 2007 through April 2008 were invited to participate in the study. Participants completed study visits at enrollment and then at 6 and 12 months.

At enrollment, data on patients’ demographic characteristics, medical history, and antimicrobial drug use within the past 12 months, and microbiologic data on previous *S. aureus* infections were obtained from electronic medical records. Participants also completed a questionnaire at enrollment and at 12 months that focused on their living situation, self-reported history of skin infections, personal hygiene, sexual behavior, and drug use over the past 12 months.

### Microbiologic Procedures

At each study visit, specimens for *S. aureus* culture were collected from the anterior nares and the groin by using sterile rayon swabs and placed in liquid Stuart’s transport media (Becton Dickinson, Sparks, MD, USA). Study staff collected specimens from the anterior nares, and participants were instructed (using a diagram of the human body) to collect specimens from the groin by swabbing in the skin folds between the thigh and genital area. Swabs were plated on mannitol salt agar (Becton Dickinson) and CHROMagar MRSA (Becton Dickinson) and then placed in 5 mL of trypticase soy broth with 6.5% sodium chloride (Becton Dickinson) as described (*19*,*20*). At each study visit, participants were classified as MRSA colonized if MRSA was detected from either the nares or groin culture. Participants were classified as colonized with methicillin-susceptible *S. aureus* (MSSA) if MSSA was detected and MRSA was not detected. Participants colonized with both MSSA and MRSA (regardless of site) were classified as MRSA colonized.

All MRSA isolates were genotyped by PFGE with *Sma*I (New England Biolabs, Beverly, MA, USA) as described (*13*,*19*,*20*). PFGE patterns were analyzed with BioNumerics Software v 5.10 (Applied Maths, Austin, TX, USA) and were assigned to USA pulsed-field types by using Dice coefficients and 80% relatedness. USA500, Iberian, and Archaic PFGE types were grouped together as USA500/Iberian because they are closely related and difficult to separate by PFGE (*21*). PCR was used to screen for staphylococcal cassette chromosome *mec* type and to detect Panton-Valentine leukocidin genes for all isolates (*22*). USA300 was defined as an isolate with a USA300 PFGE pattern that was positive for Panton-Valnetine leukocidin genes and contained staphylococcal cassette chromosome *mec* type IVa.

### Prospective Monitoring for Incident MRSA Clinical Infections

Electronic medical and microbiology records were prospectively monitored for incident MRSA clinical infections for 24 months. Participants were classified with a MRSA clinical infection if a clinical infection was documented in the medical record and MRSA was isolated from the culture. Participants with a MRSA clinical infection completed a supplemental questionnaire that focused on the signs and symptoms of their infection and its clinical course. We defined a skin and soft tissue infection in the groin as an infection that involved the buttocks, perineum, genitals, anus, or proximal thigh.

### Statistical Methods

The primary analysis compared participants in whom a MRSA clinical infection developed with those in whom a MRSA clinical infection did not develop. All analyses were performed by using SAS version 9.2 (SAS Institute Inc., Cary, NC, USA). The Wilcoxon rank-sum test (continuous variables) and the χ^2^ and Fisher exact tests (categorical variables) were used to test for differences in clinical, demographic, and behavioral variables among participants with and without MRSA clinical infection. Statistical significance was indicated by a p value <0.05. By using a multivariate log-linked binomial regression model (Proc Genmod; SAS Institute Inc.) (*23*), adjusted risk ratios (aRRs), and 95% CIs were calculated to identify variables associated independently with the development of MRSA clinical infection. All statistically significant (p<0.05) variables in univariate analysis (unadjusted RR) were included in a multivariate model (*24*), and variables with p>0.2 in the adjusted model were dropped sequentially to create a parsimonious model that was examined for goodness of fit after each step. We also evaluated variables in the final parsimonious model with Kaplan-Meier survival methods with corresponding log-rank tests and Cox proportional hazards models (Proc PHREG; SAS Institute Inc.) of time to MRSA clinical infection.

## Results

**Figure 1:**
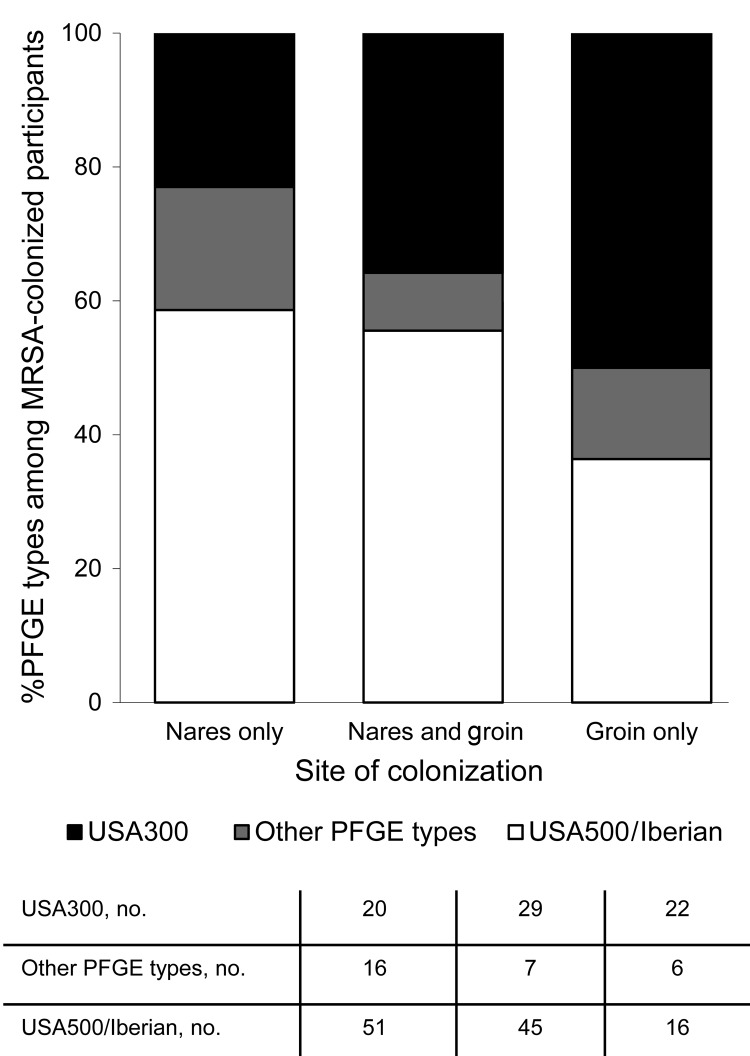
Percentage of pulsed-field gel electrophoresis (PFGE) types by anatomic site of detection in methicillin-resistant *Staphylococcus aureus* (MRSA)–colonized HIV-infected adults (n = 212 MRSA colonizing isolates; 3 study visits aggregated), Atlanta, Georgia, USA, 2007–2009.

We enrolled 600 HIV-infected veterans, most of whom (98%) were male with a median age of 52 years (interquartile range [IQR] 45–59 years). Four hundred forty-one (74%) participants were non-Hispanic Blacks, and 315 (53%) were men who had sex with men. The median most recent CD4 cell count was 416 cells/μL (IQR 250–579 cells/μL), and 474 (79%) participants were receiving antiretroviral therapy. MRSA colonization was detected in 79 (13%) of 600 participants at baseline, in 66 (13%) of 502 at 6 months, and in 62 (15%) of 426 participants at 12 months (Table 1). In addition, MRSA colonization with 2 distinct MRSA strains was detected in 2 participants at baseline, in 1 participant at 6 months, and in 2 participants at 12 months, resulting in a total of 81, 67, and 64 isolates collected at each of the 3 time points, respectively. MSSA was detected in 180 (30%) participants at baseline, 156 (31%) at 6 months, and 118 (28%) at 12 months (Table 1). USA500/Iberian (n = 112, 53%) and USA300 (n = 71, 33%) were the most common colonizing MRSA PFGE types identified. USA100 (n = 14, 7%) and other PFGE types (n = 15, 7%) were uncommon. Among MRSA-colonized participants, MRSA was detected solely in the nares of 87 (41%) participants, in both the nares and groin of 81 (38%), and only in the groin for 44 (21%) participants. USA300 accounted for 20 (23%) isolates detected solely in the nares, 29 (36%) detected in the nares and the groin, and 22 (50%) detected solely in the groin (Figure 1). Compared with other PFGE types, USA300 was more likely to be detected solely in the groin (RR = 1.9; 95% CI 1.2–3.3; p = 0.02). Including groin cultures increased the overall detection of MRSA by 26% and of USA300 by 44% compared with nasal culture alone.

**Table 1:** Prevalence of *Staphylococcus aureus* colonization among HIV-infected adults, Atlanta, Georgia, USA, 2007–2009*

*S. aureus* colonization status†	Participants, no. (%)		
	At enrollment, n = 600	At 6-mo visit, n = 502	At 12-mo visit, n = 426
MRSA	79‡ (13)	66‡ (13)	62‡ (15)
MSSA	180 (30)	156 (31)	118 (28)
No *S. aureus*	341 (57)	280 (56)	246 (58)

*MRSA, methicillin-resistant *S. aureus*; MSSA, methicillin-susceptible *S. aureus;* PFGE, pulsed-field gel electrophoresis. †Obtained from nares and groin swab specimens. ‡MRSA and MSSA co-colonization was detected in 11 participants at baseline, 10 participants at 6 mo, and 9 participants at 12 mo. For analysis these participants were classified as MRSA colonized. In addition, MRSA colonization with 2 distinct MRSA PFGE patterns was detected in 2 participants at baseline, 1 participant at 6 mo, and 2 participants at 12 mo.

Over a median of 2.1 years of follow-up, 29 MRSA clinical infections occurred in 25 participants (2.5 infections/100 person-years). Skin and soft tissue infections (n = 24) were the most common, followed by pneumonia (n = 3) and bacteremia (n = 2). Three (13%) of the skin and soft tissue infections required hospitalization, and 13 (54%) of 24 skin and soft tissue infections occurred in the groin. Of the 25 participants in whom MRSA clinical infection developed, MRSA colonization was detected at baseline in the groin only or groin and nares in 12 (48%) of 25 participants in whom a MRSA clinical infection developed, compared with 37 (6%) of 575 participants in whom an infection did not develop (p<0.0001). MRSA colonization was also detected at a study visit (baseline, 6 months, or 12 months) preceding clinical infection in 17 (68%) of 25 participants. Among clinical isolates available for PFGE typing from an initial MRSA clinical infection (n = 22), USA300 (n = 14, 64%) was the most common and was identified in 9 (69%) of 13 skin and soft tissue infections that occurred in the groin (Table 2). USA500/Iberian (n = 8, 36%) was also common and was identified in all of the pneumonia and bacteremia infections. In patients with preceding colonization, the PFGE type of the clinical isolate and preceding colonizing isolate always matched (n = 17/17).

**Table 2:** Location of infection and preceding colonization status of 25 HIV-infected adults with MRSA clinical infection, Atlanta, Georgia, USA, 2007–2009*

Infection/ participant	Infection location	Clinical infection PFGE type	Colonization status preceding clinical infection	Colonizing PFGE type	Second clinical infection
Skin and soft tissue infections in the groin					
VA1	Buttock	USA300	None	–	
VA2	Buttock	USA300	Nares and groin	USA300	
VA3	Buttock	USA300	None	–	
VA4	Perianal	USA300	None	–	
VA5	Perianal	USA300	Nares and groin	USA300	
VA6	Pubic area	USA300	Nares and groin	USA300	
VA7	Scrotum	USA300	None	–	
VA8	Thigh	USA300	None	–	
VA9	Hip	USA500/Iberian	Nares and groin	USA500/Iberian	
VA10	Rectum	USA500/Iberian	Nares and groin	USA300 and USA500/Iberian	1 mo later: USA500/Iberian rectal infection
VA11	Buttock	No specimen	Groin only	USA300	
VA12	Buttock	No specimen	Nares and groin	USA300	
Skin and soft tissue infections outside of the groin					
VA13	Axilla	USA300	Nares and groin	USA300	
VA14	Axilla	USA300	Groin only	USA300	
VA15	Lip	USA300	None	–	
VA16	Lower extremity	USA300	None	–	
VA17	Lower extremity	USA300	Nares and groin	USA300 and USA100	
VA18	Scalp	USA300	Groin only	USA300	
VA19	Back	USA500/Iberian	Nares and groin	USA500/Iberian	
VA20	Scalp	USA500/Iberian	Nares and groin	USA500/Iberian	12 mo later: USA500/Iberian decubitus ulcer infection
VA21	Scalp	No specimen	Nares and groin	USA300	
Invasive clinical infections					
VA22	Bloodstream	USA500/Iberian	Nares and groin	USA500/Iberian	
VA23	Bloodstream	USA500/Iberian	Nares and groin	USA500/Iberian	
VA24	Lung	USA500/Iberian	Nares and groin	USA500/Iberian	6 mo later: USA500/Iberian pneumonia
VA25	Lung	USA500/Iberian	None	–	2 mo later: USA500/Iberian infection on foot

*MRSA, methicillin-resistant *Staphylococcus aureus*; PFGE, pulsed-field gel electrophoresis; –, not applicable because the participant was not colonized before their clinical infection.

In univariate analysis, factors associated with an increased risk of developing MRSA clinical infection included MRSA colonization detected in the groin at baseline, a lower CD4 cell count, a previous history of an abscess, a medical history of MRSA clinical infection, renal insufficiency, a history of syphilis, the use of certain antistaphylococcal agents in the past 12 months, contact with a prison or jail, and certain hygienic factors (Table 3, appendix). A suppressed HIV viral load (<400 copies/mL) and use of antiretroviral therapy were associated with a lower risk for development of MRSA clinical infection. Of note, no MRSA clinical infections developed in the 30 participants with MRSA colonization that was detected solely in the nares at baseline. In multivariate analysis, MRSA colonization detected in the groin at baseline (aRR 4.8, 95% CI 2.1–10.8) and a medical history of MRSA clinical infection (aRR 3.195% CI 1.4–7.3) were the only 2 factors that remained significantly associated with an increased risk for development of MRSA clinical infection (Table 3, appendix). MRSA colonization detected in the groin included participants with MRSA colonization detected only in the groin (n = 14; aRR 6.6) and participants with MRSA colonization detected in the nares and groin (n = 35; aRR 4.2). This analysis was repeated by using a multiple-predictor Cox proportional hazards model to account for time to MRSA clinical infection and MRSA colonization detected in the groin at baseline (adjusted hazard ratio 5.9; 95% CI 2.5–13.9) and a medical history of MRSA clinical infection (aHR 4.0; 95% CI 1.6–9.6) were significant predictors of time to MRSA clinical infection. Among the 79 participants with MRSA colonization at baseline, USA300 colonization was associated with a nonsignificant but increased risk of developing MRSA clinical infection, compared with other PFGE types (RR 2.1; 95% CI 0.7–5.9). In a separate analysis, MSSA colonization was not associated with developing a MRSA clinical infection (RR 0.8; 95% CI 0.3–2.7).

**Table 3:** Factors associated with MRSA clinical infection among 600 HIV-infected adults, Atlanta, Georgia, USA, 2007–2009*

Factor	With MRSA clinical infection, n = 25	Without MRSA clinical infection, n = 575	Relative risk (95% CI)†	Adjusted relative risk (95% CI)
Median age, y (IQR)	50 (45–58)	52 (45–60)	p = 0.48	
Sex				
M	25 (4)	565 (96)	1.0	
F	0	10 (100)	NC	
Race/ethnicity				
Non-Hispanic Black	22 (5)	419 (95)	1.0	
Non-Hispanic White	3 (2)	147 (98)	2.5 (0.8–8.2)	
Hispanic, other, or not specified	0	9 (100)	NC	
HIV transmission risk				
MSM	14 (4)	301 (96)	1.0	
IDU	3 (4)	67 (96)	1.0 (0.3–3.3)	
MSM and IDU	0	19 (100)	NC†	
High-risk heterosexual contact‡	1 (3)	34 (97)	0.7 (0.1–4.7)	
No risk factor specified or other§	7 (4)	154 (96)	1.0 (0.4–2.5)	
CD4 cell count, median cells/μL (IQR)	340 (85–482)	419 (253–582)	p = 0.05	
CD4 cell count, cells/μL (%)				
>500	5 (2)	213 (98)	1.0	
201–500	12 (4)	257 (96)	1.9 (0.7–5.4)	
≤200	8 (7)	105 (93)	3.1 (1.0–9.2)	
HIV viral load, copies/mL (%)				
≥400	14 (7)	188 (93)	1.0	
<400	10 (3)	374 (97)	0.4 (0.2–0.9)	
MRSA baseline colonization				
Not MRSA colonized	13 (2)	508 (98)	1.0	1.0
MRSA colonized in groin	12 (24)	37 (76)	9.8 (4.7–20.3)	**4.8 (2.1–10.8)**
MRSA colonized in nares only	0	30 (100)	NC	NC
Medical history				
Abscess	11 (9)	117 (91)	2.9 (1.3–7.0)	
Cellulitis	4 (10)	36 (90)	2.9 (0.9–8.8)	
MRSA clinical infection	11 (14)	70 (86)	5.0 (2.4–10.7)	**3.1 (1.4–7.3)**
MSSA clinical infection	2 (4)	48 (96)	1.0 (0.2–3.9)	
Hospitalization in past 12 mo	5 (6)	79 (94)	1.5 (0.6–4.0)	
Diabetes	4 (6)	67 (12)	1.4 (0.5–4.0)	
Renal insufficiency	5 (10)	47 (90)	2.6 (1.0–6.7)	2.9 (0.9–9.3)
Syphilis	10 (8)	109 (92)	2.7 (1.2–5.8)	
Current medications				
Antiretroviral therapy	16 (3)	458 (97)	0.5 (0.2–1.0)	
Prescribed PCP§ prophylaxis	4 (4)	109 (96)	0.8 (0.3–2.4)	
Prescribed TMP/SMX as PCP prophylaxis	2 (3)	78 (97)	0.6 (0.1–2.4)	
Antibiotic use in past 1 y				
Any with anti–*S. aureus* activity¶	11 (4)	260 (96)	1.0 (0.4–2.1)	
Any fluoroquinolone	3 (4)	77 (96)	0.9 (0.3–2.9)	
TMP/SMX, not as PCP prophylaxis	7 (8)	78 (92)	2.4 (1.0–5.5)	
Azithromycin	5 (5)	88 (95)	1.3 (0.5–3.5)	
Vancomycin or linezolid	3 (14)	19 (86)	3.6 (1.2–11.1)	
Resided in past 12 mo				
Prison or jail	5 (12)	37 (88)	3.3 (1.3–8.4)	2.5 (0.97–6.7)
Homeless shelter	2 (5)	42 (95)	1.1 (0.3–4.5)	
Sexual behavior in past 12 mo				
Sexually active	16 (4)	368 (96)	1.0 (0.4–2.2)	
Sex with male same-sex partner	11 (4)	234 (96)	1.1 (0.5–2.4)	
Sex with ≥2 male same-sex partners	7 (5)	130 (95)	1.3 (0.5–3.0)	
Condom usage in past 12 mo				
Not sexually active	9 (4)	200 (96)	1.0	
Frequent or sometimes	14 (4)	318 (96)	1.0 (0.4–2.2)	
Rare or never	2 (3)	57 (97)	1.3 (0.3–5.7)	
Hygienic factors in past 12 mo				
Have skin contact with abscess	10 (7)	124 (93)	2.3 (1.1–5.0)	
Get bug bites	10 (4)	229 (96)	1.0 (0.5–2.2)	
Bite fingernails	5 (4)	134 (96)	0.8 (0.3–2.2)	
Pick nose	14 (6)	240 (94)	1.7 (0.8–3.8)	
Pick skin	7 (5)	123 (95)	1.4 (0.6–3.3)	
Use public hot tub or sauna	5 (7)	67 (93)	1.8 (0.7–4.7)	
Shave face	20 (4)	424 (96)	1.4 (0.5–3.7)	
Shave head	12 (8)	141 (92)	2.7 (1.3–6.4)	2.1 (0.9–4.7)
Shave chest	5 (10)	46 (90)	2.7 (1.1–6.9)	
Shave groin, genital, or buttock area	10 (9)	104 (91)	2.8 (1.3–6.2)	2.1 (0.9–6.7)
Drug use in past 12 mo				
Methamphetamine	0	20 (100)	NC	
Cocaine or crack	3 (3)	89 (97)	0.8 (0.2–2.5)	
Poppers or nitrites	3 (5)	53 (95)	1.3 (0.4–4.3)	
Injected or skin-popped any drug	0	13 (100)	NC	

*Values are no. (%) unless otherwise indicated. **Boldface** indicates significant results. MRSA, methicillin-resistant *Staphylococcus aureus*; IQR, interquartile range; NC, not calculated because there were insufficient events to calculate the relative risk; MSM, men who have sex with men; IDU, injection drug use; MSSA, methicillin-susceptible *S. aureus*; PCP, *Pneumocystis* pneumonia; TMP/SMX, trimethoprim/sulfamethoxazole.  †p values are Wilcoxon rank-sum test results. ‡Sexual contact with a person known to be HIV-infected or at high risk for HIV infection (e.g., history of IDU or MSM). §Risk not specified or unknown (n = 152); other (n = 9) indicates transfusion (n = 4) or health care worker occupational exposure (n = 5). ¶Antibiotics with anti-staphylococcal activity used were fluoroquinolones, TMP/SMX, azithromycin, clarithromycin, mupirocin, cephalexin, vancomycin, linezolid, and ampicillin-clavulanate.

**Figure 2:**
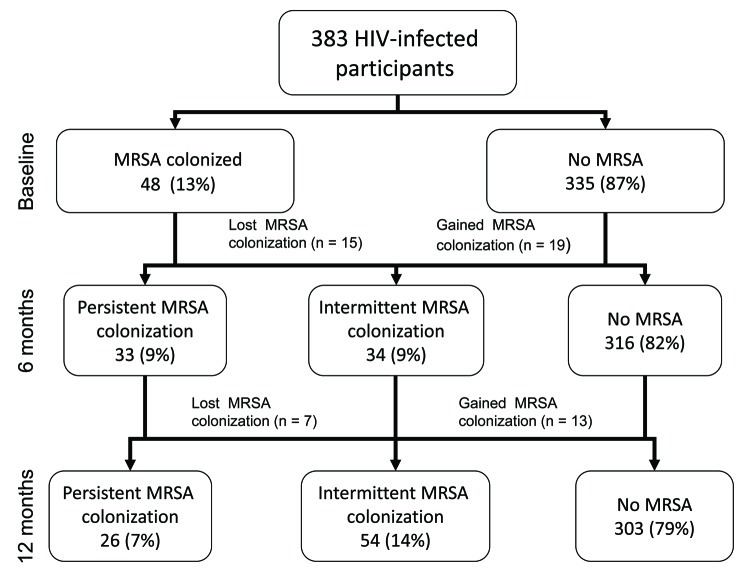
Prevalence of methicillin-resistant *Staphylococcus aureus* (MRSA) recovered from nares and groin swabs of HIV-infected adults at each study visit among participants who had specimens cultured at all 3 visits (n = 383). Atlanta, Georgia, USA, 2007–2009.

In a subanalysis of MRSA colonization in 383 HIV-infected adults from whom samples were cultured at all 3 visits, MRSA colonization was detected in 48 (13%) participants at baseline, in 52 (14%) at 6 months, and in 50 (13%) at 12 months. Approximately equal numbers of participants became colonized with MRSA or were no longer colonized at each sequential study visit to maintain this stable colonization prevalence (Figure 2). On a percentage basis at each sequential study visit, 21%–31% of MRSA-colonized participants were no longer colonized (without treatment) and 4%–6% of previously uncolonized participants became colonized with MRSA. Over 12 months, MRSA colonization was persistent (detected at all 3 visits) in 26 (7%) participants and intermittent (detected in 1 or 2 visits) in 54 (14%) participants (Figure 2). The PFGE type remained stable in 23 (88%) of 26 participants with persistent colonization and in 16 (89%) of 18 participants with intermittent colonization at 2 visits. Swab specimens from participants with persistent colonization (n = 78 MRSA isolates from 26 participants) were more likely to yield heavy growth of MRSA (growth detected on direct agar plating without broth enrichment) than were isolates from participants with intermittent colonization (n = 72 MRSA isolates from 54 participants) [91% vs. 75%; p = 0.009]. PFGE type (USA300 vs. other PFGE types) was not significantly associated with persistent vs. intermittent colonization (p = 0.27).

## Discussion

MRSA clinical infections (mainly skin and soft tissue infections) were common among HIV-infected adults in this study. The prevalence of MRSA colonization was also high at each study visit (13%–15%), and MRSA colonization in the groin was a risk factor for developing a MRSA clinical infection. MRSA PFGE types USA300 and USA500/Iberian were common causes of colonization and clinical infection. USA300 more commonly caused colonization of the groin and clinical skin and soft-tissue infection in the groin. MRSA prevention strategies with HIV-infected adults that can effectively address colonization at this anatomical site are likely necessary to reduce MRSA clinical infections in this population.

HIV-infected persons have been found to have 6× the risk for community-associated MRSA skin and soft-tissue infections than HIV-negative patients (*25*) and an increased odds of having community-acquired *S. aureus* bacteremia (*26*). In this study, MRSA colonization in the groin and a medical history of MRSA clinical infection were risk factors for clinical infection. Because, in our study, most skin and soft tissue infections occurred in the groin, colonization of the groin may have directly precipitated clinical infection in this anatomical area. In a previous analysis, we demonstrated that MRSA colonization was also associated with a medical history of MRSA clinical infection, contact with jails and prisons, and correlates of risky sexual behavior (i.e., rarely or never using condoms) (*20*). In addition, in this analysis, adjusting for MRSA colonization in the groin diminished the association between MRSA clinical infection and risk factors for exposure to MRSA (e.g., contact with jails and prisons) and risk factors related to hygiene (e.g., shaving the groin, genital, or buttock area). These findings suggest that MRSA colonization in the groin may also be a marker of more frequent exposure to MRSA in the environment or poor hygiene or an indicator of immunologic dysfunction (i.e., impaired neutrophil function [*27*]) that in turn increases a person’s susceptibility to clinical infection.

Prior studies have demonstrated that USA300 causes most community-associated MRSA infections in the United States, whereas USA500/Iberian clones are associated with health care–associated MRSA infections (*1*). This epidemiology, however, is changing (*28*), and participants in this study had risk factors for both community-associated and outpatient health care–associated MRSA exposures. In this study, USA300 caused most skin and soft tissue infections and was more likely colonize the groin only. Other PFGE types, however, also caused both clinical infections and groin colonization, and PFGE type (USA300 vs. other PFGE types) was not independently associated with risk for MRSA clinical infection. These findings suggest that the presence of MRSA colonization in the groin is more useful clinical knowledge than identifying the PFGE type causing colonization (which is rarely determined in clinical practice anyway).

The association of MRSA colonization and the development of clinical infection in this study suggest that MRSA decolonization with topical or systemic treatment may be an effective method to prevent clinical infections in this population. A randomized clinical trial of adult hospitalized surgical patients found that using intranasal mupirocin and chlorhexidine gluconate soap total-body wash substantially reduced the rate of health care–associated *S. aureus* infection by 58% in patients who were nasal carriers of *S. aureus* (*29*). Although several randomized controlled trials have demonstrated that MRSA colonization can be eliminated from the groin (*30*), and short-term clinical benefits of *S. aureus* decolonization have been demonstrated in the hospitalized setting, data have not been available to support decolonization as a method of preventing MRSA clinical infections in a community or outpatient setting (*31*). In this study, we observed that although MRSA colonization was frequent, it also fluctuated considerably over time. MRSA colonization spontaneously resolved in approximately half of participants over 12 months, but new MRSA colonization was detected in as many previously uncolonized participants. A MRSA decolonization program would therefore treat a substantial number of persons whose MRSA colonization would have resolved spontaneously and would require ongoing screening to identify new colonization. The substantial fluctuations in MRSA colonization status in this study suggest that strategies that emphasize hygiene and avoidance of potential MRSA exposures might be more effective at preventing MRSA clinical infections in this setting than decolonization, but this hypothesis should be tested in a clinical study that includes decolonization of MRSA from the groin as an intervention.

Our study had several limitations. First, our study population was 98% male and our findings are not generalizable to HIV-infected women. Second, the MRSA epidemic in the United States continues to evolve (*7*), and new risk factors for MRSA infection in HIV-infected adults may emerge that were not significant in this study. In addition, in our study, some risk factors for community-associated MRSA clinical infections, such as methamphetamine use (*9*) and close contact with someone with a skin infection, were not associated with MRSA clinical infection. These differences might be explained by low frequencies of certain risk factors (i.e., methamphetamine use) in our study population or a social desirability bias may have limited the full disclosure of drug use and sexual and hygienic behavior. Third, we evaluated 45 variables in univariate analysis and 16 variables in the initial multivariate model before creating a final parsimonious model with 6 variables. Although evaluating an extensive list of potential risk factors for MRSA clinical infection had some advantages, the extensive list also increased variance in the initial multivariate model. Fourth, the optimal sampling (i.e., which sites to swab and how to collect the specimen) and microbiologic techniques to evaluate MRSA colonization in the groin have not been established. Although we used microbiologic techniques that have been demonstrated to improve MRSA detection (*19*), we may have underestimated the true prevalence of groin colonization. Finally, participants may have had MRSA clinical infections that were not cultured, and these infections would not have been captured by our electronic monitoring of microbiologic records. Therefore, we might have underestimated the true incidence of MRSA clinical infections in this population.

In this study of HIV-infected adults, MRSA clinical infections were common and associated with MRSA colonization in the groin and a medical history of MRSA clinical infection. MRSA PFGE types USA300 and USA500/Iberian contributed to clinical infections, and participants had risk factors for both community-associated and health care–associated MRSA exposures. Given this high incidence of MRSA clinical infections, both community-associated and hospital-associated MRSA prevention strategies should be emphasized in HIV-infected adults in settings with high rates of MRSA clinical infections. Current community-associated MRSA prevention strategies include keeping cuts and scrapes clean and covered; practicing good hand hygiene; avoiding shared personal items, such as towels and razors; and decolonization in certain situations (*31*). Given the frequency of MRSA colonization in the groin and its association with clinical infection, MRSA prevention strategies (both hygienic practices and decolonization treatments) with HIV-infected adults should be used to prevent or eliminate colonization at this anatomic site to reduce MRSA clinical infections in this population.
